# High- and Low-Affinity Epidermal Growth Factor Receptor-Ligand Interactions Activate Distinct Signaling Pathways

**DOI:** 10.1371/journal.pone.0015945

**Published:** 2011-01-10

**Authors:** Jordan A. Krall, Elsa M. Beyer, Gavin MacBeath

**Affiliations:** 1 Department of Chemistry and Chemical Biology, Harvard University, Cambridge, Massachusetts, United States of America; 2 Department of Molecular and Cellular Biology, Harvard University, Cambridge, Massachusetts, United States of America; 3 Department of Systems Biology, Harvard Medical School, Boston, Massachusetts, United States of America; University of Birmingham, United Kingdom

## Abstract

Signaling mediated by the Epidermal Growth Factor Receptor (EGFR) is crucial in normal development, and aberrant EGFR signaling has been implicated in a wide variety of cancers. Here we find that the high- and low-affinity interactions between EGFR and its ligands activate different signaling pathways. While high-affinity ligand binding is sufficient for activation of most canonical signaling pathways, low-affinity binding is required for the activation of the Signal transducers and activators of transcription (Stats) and Phospholipase C-gamma 1 (PLCγ1). As the Stat proteins are involved in many cellular responses including proliferation, migration and apoptosis, these results assign a function to low-affinity interactions that has been omitted from computational models of EGFR signaling. The existence of receptors with distinct signaling properties provides a way for EGFR to respond to different concentrations of the same ligand in qualitatively different ways.

## Introduction

EGFR is a member of the receptor tyrosine kinase family, which functions to sense and respond to extracellular signals. Ligand binding to the extracellular domain of EGFR induces receptor dimerization, activation of its kinase domain, and phosphorylation of tyrosine residues in its carboxy terminal tail [1]. Intracellular proteins containing Src homology 2 (SH2) or phosphotyrosine binding (PTB) domains bind to these sites of tyrosine phosphorylation [2], initiating a wide variety of signaling cascades including the Ras/MAPK, PI3K/Akt, PLCγ/PKC, and Stat pathways [3]. These signals induce diverse cellular responses, including proliferation, differentiation, migration, survival, and apoptosis.

Scatchard analysis has shown that EGFR binds its ligands with two distinct affinities and has been thought to indicate the presence of two distinct populations of receptor [4]. High-affinity receptors (*K*_D_≈300 pM) generally constitute ∼10% of the total receptor pool, and low-affinity receptors (*K*_D_≈2 nM) constitute the remaining ∼90% [5]. These receptors are thought to be derived from the same transcript and to be identical in amino acid sequence. It has been suggested that the difference in affinity arises from differential localization of the receptor in the plasma membrane [6] or interaction with an “external site”, such as coated-pits [7]. More recently, Macdonald and Pike have presented evidence that high- and low-affinity binding can be explained by negative cooperativity, rather than by the existence of two distinct populations of receptor [8]. Their model predicts that at low concentrations of ligand, singly occupied dimers will be most abundant, whereas at high concentrations of ligand, doubly occupied dimers are also present.

Early reports suggested that most, if not all, cellular responses to EGFR ligands could be generated by signaling through the small percentage of high-affinity receptors [9], [10]. The fact that very high ligand concentrations induce different phenotypes [11]–[16], however, suggests that low-affinity binding may also play an important role in signaling. Here we demonstrate that high- and low-affinity interactions between EGFR and its ligands activate distinct signaling pathways and, further, that the low-affinity interactions play a crucial role in determining cellular outcome. We find that most intracellular signaling pathways, including the Ras/MAPK and PI3K/Akt pathways, are activated upon stimulation of cells with extremely low concentrations of Epidermal Growth Factor (EGF) or Transforming Growth Factor-alpha (TGFα). In contrast, certain proteins, including the Stat transcription factors, cannot be activated unless a higher concentration of ligand is used. The concentration of ligand required to activate the Stat proteins coincides with the low-affinity interaction of EGFR. Moreover, it coincides with a change in the phenotypic outcome of stimulated cells. Whether high- and low-affinity receptors are distinct populations or differ only in their ligand occupancy, our data argue that high- and low-affinity receptors mediate distinct biological processes, allowing EGFR to induce qualitatively different responses to the same ligand. Additionally, these findings should inform future efforts to model the EGFR signaling network and enhance computational approaches aimed at predicting cell decision processes mediated by this receptor.

## Materials and Methods

### Cell Culture

A431, HMEC and MDA-MB-468 cells were obtained from ATCC (Manassas, VA). The generation of HEK293-EGFR cells (HEK Flp-In-293 cells expressing ectopic EGFR) has been described [17]. A431, MDA-MB-468 and HEK293-EGFR cells were maintained in Dulbecco's Modified Eagle Medium (DMEM; Mediatech; Herndon, VA) supplemented with 10% (v/v) Fetal Bovine Serum (FBS; Hyclone; Logan, UT), 2 mM Glutamine, 100 I.U./mL Penicillin, 100 µg/mL Streptomycin (all from Mediatech). Culture medium for HEK293-EGFR cells also included 150 µg/mL Hygromycin B (Invitrogen; Carlsbad, CA). HMECs were maintained in HuMEC Basal Serum-free Medium containing the HuMEC supplement and bovine pituitary extract (Invitrogen). Unless otherwise indicated, cells were serum-starved for 24 hours before all experiments. For HMECs, serum starvation is defined as culture in HuMEC Basal Serum-free Medium without the addition of supplements. To determine cell number, cells were treated with trypsin, resuspended in PBS, and counted using a Cellometer® AutoT4 (Nexcelom Biosciences; Lawrence, MA).

### Lysates and Immunoblotting

Cells were stimulated with EGF (Millipore; Billerica, VA) or TGFα (PeproTech; Rocky Hill, NJ) at the indicated concentrations. After treatment with growth factors, cells were washed twice with cold PBS and lysed by adding 0.5 mL of lysis buffer [50 mM Tris-HCl, 1% NP-40 (v/v), 5 mM EDTA, 1 mM NaF, pH 8.0 supplemented with 10 mM β-glycerol phosphate, 1 mM phenylmethanesulfonyl fluoride, 1 mM sodium orthovanadate, 1% Phosphatase Inhibitor Cocktail II (Sigma; St. Louis, MO), and 1 Complete-Mini Protease Inhibitor Cocktail tablet (Roche Applied Science; Indianapolis, IN) per 10 mL]. Lysates were cleared by centrifugation at 20,000 *g*, 15 min, 4°C. Total protein concentration was determined using the MicroBCA Protein Assay (Pierce Biotechnology; Rockford, IL). Prior to immunoblotting, lysates were boiled in standard SDS gel-loading buffer and loaded onto 8, 10, or 12% polyacrylamide gels (10 µL lysate per lane). After separation by electrophoresis, the proteins were transferred to nitrocellulose and the membranes were blocked with 5% nonfat dry milk (w/v) in Tris-buffered saline (20 mM Tris, 150 mM NaCl, pH 7.6) containing 0.1% Tween-20 (v/v). Membranes were probed using rabbit-derived primary antibodies from Cell Signaling Technologies (Beverly, MA): Akt pS473 (catalogue number: 9271), Cbl pY774 (3555), CrkL pY207 (3181), Erk pT202/pY204 (4377), Gab1 pY307 (3234), PLCγ1 pY783 (2821), Shc1 pY239/240 (2434), SHP-2 pY542 (3751), Src pY416 (2101), Stat1 pY701 (9167), Stat3 pY705 (9131), Stat5 pY694 (9351). Bands were detected with IRDye 680-labelled goat-anti-rabbit IgG (LI-COR Biosciences; Lincoln, NE) and imaged using an Odyssey Infrared Imaging System (LI-COR Biosciences). The intensity of each band was quantified and then normalized based on the protein concentration of the lysate.

### EGF binding curves

Serum-starved cells or cells growing in 10% serum were treated with trypsin and washed in PBS. Cells were incubated for 5 h at 4°C in medium containing 0.5% BSA and Alexa Fluor 488-labeled EGF (Invitrogen) at the indicated concentration. Cells were then washed, resuspended in 0.5% BSA in PBS, and passed through 0.7-µm cell strainers. Mean fluorescence intensity was measured by flow cytometry using an LSRII flow cytometer (BD Biosciences).

### BrdU incorporation assays

Serum-starved cells or cells growing in 10% serum were treated with EGF for the indicated time. One hour prior to the end of the incubation, BrdU (BD Biosciences; Franklin Lakes, NJ) was added to the culture medium to a final concentration of 20 µg/mL. After a 1-hour incubation, cells were washed twice in cold PBS and treated with trypsin (Mediatech). Cells were washed twice with room temperature PBS, resuspended in cold 70% ethanol, and stored at 4°C in the dark for 2–5 days. After ethanol fixation, cells were resuspended in 1 mL cold 0.1% Triton X-100, 0.1 M HCl and incubated on ice for 1 min. Cells were washed in 5 mL of room temperature denaturation buffer (150 µM sodium chloride, 15 µM sodium citrate), resuspended in 1 mL of denaturation buffer, and heated at 95°C for 5 min. After cooling on ice for 5 min, the cells were resuspended and added to 5 mL of antibody dilution buffer (0.1% Triton X-100, 1% bovine serum albumin in PBS). After centrifugation, cells were stained with FITC-conjugated anti-BrdU antibody (BD Biosciences) in antibody dilution buffer and incubated at room temperature for 30 min. The cells were washed twice with antibody dilution buffer, resuspended in 0.5% BSA in PBS, and passed through 0.7-µm cell strainers. BrdU incorporation was assessed by flow cytometry using an LSRII flow cytometer (BD Biosciences).

### Phase-contrast imaging

A431 cells were plated in 6-well plates and either grown in 10% serum or serum-starved prior to a 12-hour treatment with EGF. Phase contrast images were generated using an Axiovert 200 microscope (Zeiss; Thornwood, NY) equipped with an environmental chamber (Solent; Segensworth, United Kingdom) that was held at 37°C throughout all experiments. Images were acquired with an Orca ERII camera in the high precision (14-bit) mode, cooled to −60°C.

## Results

### Different concentrations of EGFR ligands induce activation of distinct signaling pathways

**Figure 1:**
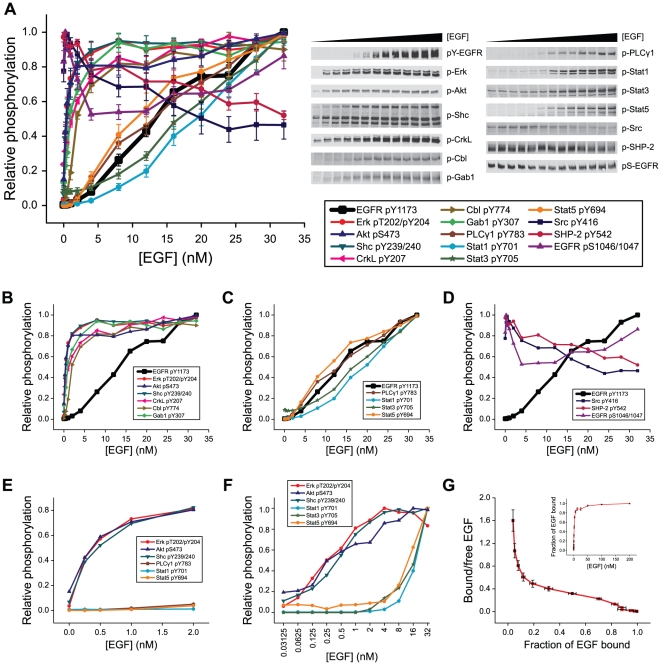
Distinct subsets of signaling proteins are activated by different concentrations of EGF. *A*–*E.* Serum-starved A431 cells were treated for five minutes with different concentrations of EGF, ranging from 250 pM to 32 nM. Phosphorylation levels were determined by immunoblotting with phosphospecific antibodies and scaled relative to the maximum level observed for each antibody. *A*. All 12 signaling proteins, as well as two sites of phosphorylation on EGFR. Error bars indicate the standard error of the mean (SEM) of three biological replicates. Representative immunoblots are shown for each antibody. *B–D.* Proteins shown in panel *A* were divided into three subsets. EGFR tyrosine phosphorylation is shown in each plot for comparison. Error bars have been omitted for clarity. *B*. Proteins that are phosphorylated at low concentrations of EGF. *C*. Proteins that require high concentrations of EGF to be phosphorylated. *D*. Proteins with atypical responses. *E*. A subset of the data from panel *A* is shown, highlighting the lowest concentrations of EGF. *F.* Serum-starved A431 cells were treated for five minutes with different concentrations of EGF, ranging from 31 pM to 32 nM. Phosphorylation levels were plotted on a log scale to illustrate responses at low EGF concentrations. *G*. A saturation-binding curve (inset) was generated for EGF binding to A431 cells. Bound EGF is scaled relative to maximum binding. A Scatchard plot of EGF binding to A431 cells was generated by plotting the ratio of bound-to-free EGF as a function of bound EGF. Error bars indicate the SEM of five biological replicates.

To explore how different ligand concentrations affect the activation of EGFR-dependent signaling pathways, we stimulated serum-starved A431 epidermoid carcinoma cells for five minutes with twelve concentrations of EGF, ranging from 250 pM to 32 nM. This early time point was chosen because the phosphorylation of many signaling proteins peaks within the first ten minutes of stimulation and because we wanted to capture early, receptor-dependent signaling events. The activation of signaling proteins was monitored by quantitative immunoblotting using phosphospecific primary antibodies followed by secondary antibodies labeled with infrared fluorophores. Tyrosine phosphorylation of EGFR, which is a marker for its enzymatic activity, was found to increase linearly with increasing EGF concentrations and did not show evidence of saturation even when cells were treated with 32 nM EGF (Figure 1A). We examined five sites of tyrosine phosphorylation on EGFR and found that they all behaved similarly (data not shown).

Downstream of receptor phosphorylation, we examined the phosphorylation levels of twelve diverse cytoplasmic signaling proteins that are activated by EGFR. For each of these proteins (Erk, Akt, Shc1, CrkL, Cbl, Gab1, PLCγ1, Stat1, Stat3, Stat5, Src and SHP-2), phosphorylation either regulates its activity or modulates its association with other signaling proteins. Because antibodies vary in sensitivity, we plotted dose-response curves as a fraction of the maximal phosphorylation observed for each protein (Figure 1A). Although we expected to observe differences in the dose-response curves as signaling proteins vary in their intracellular concentrations and molecular interactions, we found that most of the phosphorylation events fell into only two categories: phosphorylation induced by low ligand concentrations (Figure 1B) and phosphorylation induced only by high ligand concentrations (Figure 1C). The phosphorylation patterns of two cytoplasmic proteins, SHP-2 and Src, as well as of the receptor at Ser1046/1047, did not fall into either category (Figure 1D).

The set of proteins comprising Erk, Akt, Shc1, CrkL, Cbl, and Gab1 was phosphorylated in response to very low concentrations of EGF. These proteins all showed increased phosphorylation at even the lowest concentration of EGF and near maximal phosphorylation at 1 nM EGF. This response was seen both for the downstream protein Erk, whose activation does not depend on direct interaction with the receptor, and for the upstream protein Shc1, which binds directly to the receptor. Thus, the response of proteins to low concentrations of EGF does not require signal amplification downstream of receptor activation. In contrast, the set of proteins comprising Stat1, Stat3, Stat5 and PLCγ1 required higher levels of EGF for activation. At EGF concentrations below 1 nM, there was no detectable induction of phosphorylation of these proteins (Figure 1, E and F). At higher concentrations, phosphorylation increased linearly and tracked closely with the phosphorylation of EGFR on its intracellular tyrosine residues. Importantly, basal phosphorylation levels of these proteins were detectable, indicating that the antibodies we used were sensitive enough to reliably determine the lowest concentration of EGF at which phosphorylation increased.

**Figure 2:**
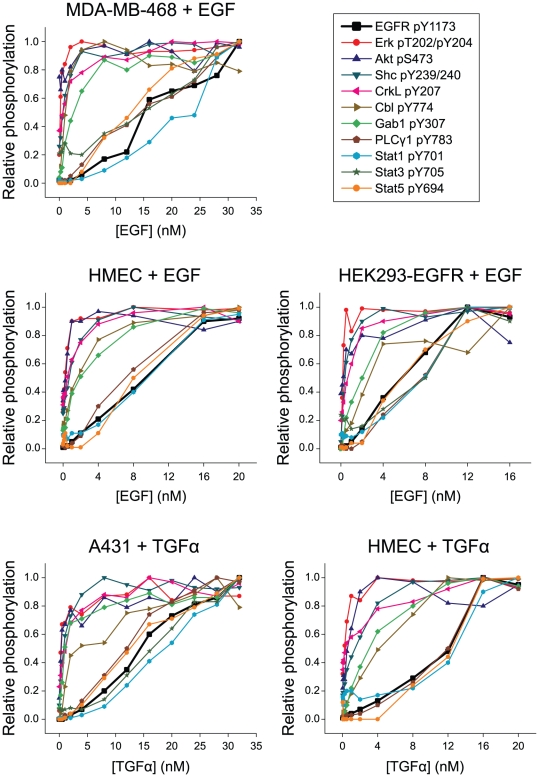
Distinct subsets of signaling proteins are activated by different concentrations of both EGF and TGFα in multiple cell lines. Serum-starved cells were treated for five minutes with different concentrations of EGF or TGFα, ranging from 250 pM to 32 nM. Phosphorylation levels were determined by immunoblotting with phosphospecific antibodies and scaled relative to the maximum level observed for each antibody.

To determine if the signaling responses that we observed in A431 cells were cell line-specific, we examined concentration-dependent signaling outcomes in three additional cell lines: MDA-MB-468 breast cancer cells, which, like A431, over-express EGFR (∼10^6^ receptors per cell) [18], [19]; normal human mammary epithelial cells (HMECs; ∼10^5^ receptors per cell) [20]; and human embryonic kidney cells that were stably transfected with EGFR (HEK293-EGFR; ∼10^5^ receptors per cell) [17]. Stimulation of these three cell lines with different concentrations of EGF revealed that, in each case, the twelve intracellular signaling proteins could be divided into the same two groups that respond to either high or low concentrations of growth factor (Figure 2). We additionally found that this outcome is not growth factor-specific, as treatment of A431 or HMEC cells with different concentrations of TGFα yielded nearly identical results (Figure 2). Together these observations suggest that there are intrinsic differences in the receptors that activate these two sets of proteins, and that these differences are independent of tissue of origin, transformation, or receptor expression level.

### Low-affinity EGF binding is required for PLCγ1 and Stat protein activation

**Figure 3:**
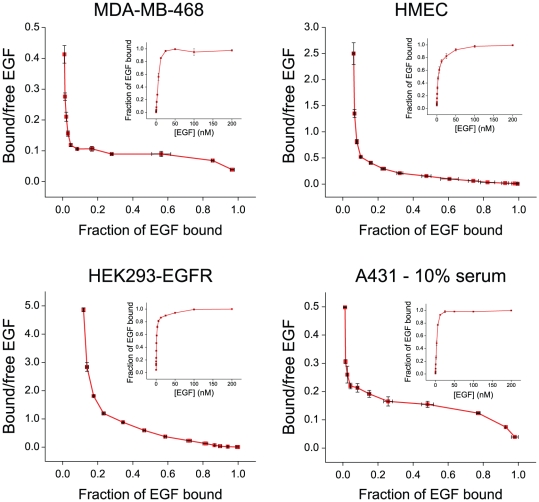
Cell lines used in this study express receptors with two distinct binding affinities. Saturation binding curves (insets) were generated for EGF binding to each cell line. Bound EGF is scaled relative to maximum binding. Scatchard plots of EGF binding were generated by plotting the ratio of bound-to-free EGF as a function of bound EGF. Error bars indicate the range of two biological replicates for MDA-MB-468, HEK293-EGFR, and A431 cells and the SEM of three biological replicates for HMECs.

The affinities and relative proportions of high- and low-affinity receptors that have previously been reported suggest their involvement in activating the two subsets of signaling proteins that we observed in our studies. To determine if the cell lines we used express receptors with two distinct binding affinities, we measured the direct binding of fluorescently labeled EGF to these cells and performed Scatchard analysis. The resulting curvilinear plots (Figure 1G and Figure 3) are characteristic of cells expressing both high- and low-affinity EGF receptors, and the ratio and affinities of these binding sites are consistent with previous reports [5]. These results suggest that many signaling proteins, including Erk, Akt, Shc1, CrkL, Cbl and Gab1, can be activated by high-affinity receptors, whereas others, including PLCγ1 and the Stat proteins, are only activated by low-affinity receptors. Because high- and low-affinity receptors are derived from the same transcript, it is not possible to selectively mutate or knock down one population or the other. Additionally, although antibodies that selectively block either high- or low-affinity binding have been reported [9], [10], in our hands they either activated the receptor or blocked all ligand binding. We were therefore unable to selectively perturb one class of receptor to further demonstrate its distinct signaling properties.

**Figure 4:**
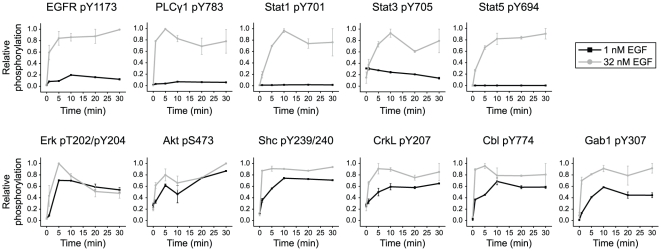
Stat proteins and PLCγ1 cannot be activated by low EGF concentrations in A431 cells. Serum-starved A431 cells were treated with either 1 nM or 32 nM EGF. Phosphorylation of EGFR and downstream signaling proteins was monitored over the course of 30 minutes by quantitative immunoblotting. PLCγ1 and the Stat proteins were only activated at the high concentration of EGF (32 nM). All other signaling proteins were activated at both high (32 nM) and low (1 nM) concentrations of EGF. Phosphorylation levels were scaled relative to the maximum signal observed for each antibody. Error bars indicate the range of two biological replicates.

We considered the possibility that PLCγ1 and the Stats may have slower rates of pathway activation than other signaling proteins and are therefore not phosphorylated in response to low concentrations of EGF. For example, at low EGF concentrations, Shc1 might simply out-compete Stat1 for the limited number of binding sites on activated high-affinity receptors, leading to a negligible increase in Stat1 phosphorylation after just five minutes of stimulation. Over time, however, phosphorylated Stat1 could accumulate as Stat1 slowly gains access to binding sites on EGFR. To test this alternative hypothesis, we monitored the phosphorylation of cytoplasmic proteins in A431 cells over 30 minutes in response to either low (1 nM) or high (32 nM) concentrations of EGF. As anticipated, the intensities of Shc1, Erk, and Akt phosphorylation were comparable at both concentrations of EGF (Figure 4). In contrast, whereas high concentrations of EGF induced robust phosphorylation of PLCγ1 and the Stat proteins, no increase in phosphorylation of these proteins was observed at low EGF levels, even after 30 minutes of stimulation (Figure 4). There was even a slight decrease in the basal level of Stat3 phosphorylation, which is likely due to the induction of phosphatases at low concentrations of EGF. Importantly, at 1 nM EGF, the activity of EGFR, as determined by receptor phosphorylation at Tyr1173, was approximately 10% of its activity at 32 nM EGF (Figure 4), demonstrating that this site is phosphorylated on both high- and low-affinity receptors. These data are inconsistent with a kinetic explanation and suggest that the phosphorylation of PLCγ1 and the Stat proteins can only be induced when low-affinity EGF receptors are activated by high concentrations of EGF. Time-course experiments in HMECs gave similar results, although signaling was downregulated more rapidly in these cells (which do not overexpress EGFR) (data not shown).

Some of the cell lines used in this study are known to maintain autocrine loops that activate EGFR, creating a steady-state level of signaling through EGFR [21]. This signaling likely occurs through both high- and low-affinity receptors, due to the high effective concentration of locally produced ligand. The addition of low levels of exogenous ligand allows for activation of primarily high-affinity receptors, while adding higher concentrations of ligand engages low-affinity receptors as well. Because autocrine signaling does not appear to saturate signaling through either receptor, the effects induced by exogenous ligand highlight the different functions of high- and low-affinity receptors even when low levels of basal signaling are present.

### Low-affinity EGFR induces distinct proliferative responses

EGF was initially identified as a secreted factor that promotes cellular proliferation. In many cell lines, however, high levels of EGF have been shown to inhibit proliferation [14]. Depending on the study, high concentrations of EGF have also been reported to induce apoptosis [11], cell cycle arrest [12], anoikis [16], or morphological changes [13] in A431 and MDA-MB-468 cells. EGF-induced apoptosis has also been observed in MCF-7 cells, which have substantially lower levels of EGFR (∼10^4^ receptors per cell), indicating that anti-proliferative responses are not unique to cells expressing high levels of EGFR [15].

**Figure 5:**
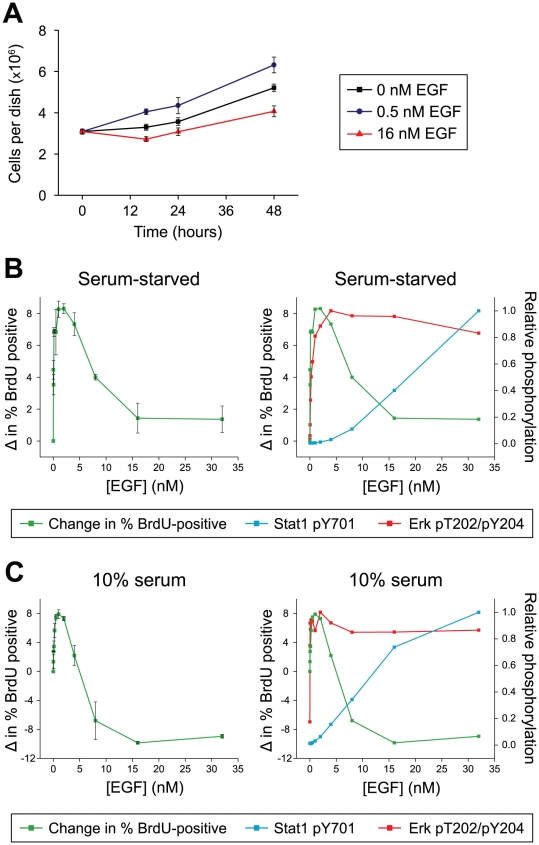
High and low concentrations of EGF induce distinct phenotypic outcomes. *A*. Serum-starved A431 cells were treated with 0 nM, 0.5 nM or 16 nM EGF. At the indicated times, cells were trypsinized and counted. Error bars represent the SEM of three biological replicates. *B.* Left, A431 cells were serum-starved for 24 hours and treated with different concentrations of EGF for 12 hours. BrdU was added to the culture medium for the last hour of the EGF incubation. Cell proliferation was recorded as the change in the percentage of BrdU-positive cells relative to unstimulated cells (no EGF). Error bars represent the SEM of three biological replicates. Right, the cell proliferation data overlaid with the relative phosphorylation levels of Erk and Stat1 as reported in Fig. 1. Error bars have been omitted for clarity. The decrease in proliferation coincides with the increase in Stat1 phosphorylation. *C.* Left, A431 cells grown in 10% serum were treated with different concentrations of EGF for 24 hours and BrdU incorporation was determined as in *B*. Right, the cell proliferation data overlaid with the relative phosphorylation levels of Erk and Stat1 in these cells. Error bars have been omitted for clarity.

In order to uncover the roles of high- and low-affinity interactions in promoting these phenotypes, we investigated the response of A431 cells to different concentrations of EGF. Cells were treated with either 0.5 nM or 16 nM EGF and counted over a three-day period. After 16 hours, treatment with 16 nM EGF resulted in a reduction in cell number, whereas treatment with 0.5 nM EGF induced cell proliferation (Figure 5A). At longer time points, cells treated with 16 nM EGF began to increase in number, but the total number of cells remained lower than was observed in serum-free medium or in 0.5 nM EGF. We observed no evidence of EGF-induced apoptosis under these conditions (data now shown).

To investigate more precisely the role of high- and low-affinity EGF receptors in cell proliferation, we monitored BrdU incorporation after treating A431 cells for twelve hours with different concentrations of EGF. Cell proliferation increased in a dose-dependent manner for concentrations up to and including 0.5 nM EGF (Figure 5B). This increase corresponded with a sharp rise in Erk phosphorylation, which is representative of signaling by high-affinity receptors (Figure 5B). The rise in BrdU incorporation leveled-off between 1 and 2 nM EGF and then decreased in a dose-dependent manner at higher concentrations. This decrease in DNA synthesis coincided with the onset of Stat1 phosphorylation, which is representative of signaling by low-affinity receptors and is thought to play an important role in the anti-proliferative effects of EGF (Figure 5B) [12]. To determine if our results can be generalized to more heterogeneous extracellular environments, we repeated our EGF stimulations using cells grown in media containing 10% serum. We found that both the binding and signaling data were almost identical to data generated under serum-free conditions (Figure 3 and data not shown). Further, when BrdU incorporation assays were performed with different concentrations of EGF, we observed a proliferative response that mirrored the response in serum-free medium (Figure 5C). Notably, at higher ligand concentrations, the observed anti-proliferative response was even more pronounced than when the cells were grown under serum-free conditions, as the levels of BrdU incorporation dropped well below basal levels. Receptor downregulaton might contribute to the observed inhibition of proliferation at high concentrations of EGF. However, the activation of Stat1 exclusively by low-affinity receptors at short time points (Figure 1) before downregulation occurs, combined with the known role of Stat1 in mediating EGF-induced growth inhibition [12], [22], argues that specific signaling events in response to high EGF concentrations induce the negative effect on proliferation. These results demonstrate that signaling by high-affinity EGF receptors favors cell proliferation, whereas signaling by low-affinity EGF receptors inhibits this response, not by decreasing signaling through Erk, but by eliciting a distinct set of opposing signals.

### Low-affinity EGFR alters cellular adhesion properties

**Figure 6:**
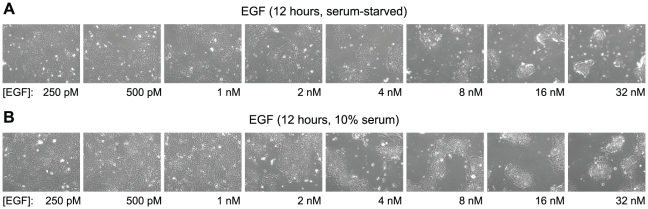
Activation of low-affinity EGFR alters cellular adhesion properties. Phase-contrast images of A431 cells treated with different concentrations of EGF for 12 hours. *A.* Serum-starved cells. *B.* Cells grown in 10% serum. Onset of the cell clumping phenotype coincides with Stat phosphorylation.

In addition to the anti-proliferative effects, we found that the collective morphology of A431 cells was altered at EGF concentrations that engage low-affinity receptors. At low concentrations of EGF, cultured cells proliferated and formed a confluent monolayer. Strikingly, at concentrations of EGF above 2 nM, cells clustered into three-dimensional islands and grew on top of each other (Figure 6A). This adhesion response was observed within two hours of adding EGF, and the effect became more pronounced with increasing EGF concentrations, mirroring the concentration-dependent decrease in cell proliferation. Cell clustering was observed both when the cells were grown under serum-free conditions (Figure 6A) and when they were grown in 10% serum (Figure 6B). Changes in integrin levels, which can be regulated by Stat3 [23], have previously been implicated in cell clustering [13]. The EGF concentrations at which the clustered morphology was induced and the potential involvement of Stat3 argue that, in addition to inhibiting cell proliferation, low-affinity receptors also mediate changes in cell adhesion properties.

## Discussion

Our studies provide evidence that low-affinity EGF receptors play crucial roles in cell-fate decisions. EGF titrations demonstrate the existence of two distinct sets of cytoplasmic signaling proteins: one that is activated by low concentrations of EGF when only a small fraction of EGFR molecules are active, and a second, comprising PLCγ1, Stat1, Stat3 and Stat5, that is only activated by higher concentrations of EGF. These results, in conjunction with the observed affinities and ratios of high- and low-affinity receptors, strongly suggest that although many signaling pathways can be activated by high-affinity EGF receptors, PLCγ1 and the Stat proteins depend on low-affinity receptors for their activation. In addition to the concentration-dependent signaling profiles, changes in cell morphology and rates of proliferation coincide with the activation of low-affinity receptors, supporting a role for these receptors in determining phenotypic outcome. Some of the cell lines used in this study maintain autocrine loops that activate EGFR even under serum-starved conditions. While this prohibits the complete elimination of negative feedback loops, the experimental setup using exogenous ligand demonstrates that even in the presence of a basal level of signaling, EGF receptors have distinct functions that are based on their ligand affinity. Accordingly, our data are very similar when cells are grown in the presence of serum, demonstrating that our findings hold true even in a more complex signaling environment.

It has been known for some time that cells exhibit different phenotypic responses to high and low concentrations of EGF. It is also well known that EGFR appears to exist in two different forms: a high-affinity form with an apparent *K*_D_ of ≈300 pM, and a low affinity form with an apparent *K*_D_ of ≈2 nM. To date, however, these two observations have not been linked to signaling outputs, as high- and low-affinity EGF receptors have never been shown to elicit specific and distinct intracellular signaling events. Here, we provide evidence that low-affinity EGF receptors activate a distinct set of intracellular signaling proteins (the Stat proteins and PLC-γ) and that the concentration of EGF or TGFα at which this occurs coincides precisely with the point at which different phenotypic outcomes are observed. To date, all mathematical models of EGFR signaling assume that the activated receptor exists in a single form and turns on a specific set of signaling proteins. These models are unable to predict the different phenotypic outcomes that are observed at different ligand concentrations. Our study provides evidence that low-affinity receptors turn on specific and distinct signaling pathways and argues strongly that predictive models of EGFR signaling should take low-affinity receptors into account.

While we would have liked to inhibit each form of the receptor independently, unfortunately there is currently no way to selectively perturb either the high- or low-affinity population of receptors, as they are both encoded by the same transcript. As there was no way to selectively perturb either population, we relied on extremely rigorous and quantitative analyses to observe coincidence between the three phenomena we were studying: ligand binding, activation of signaling proteins, and phenotypic outcome. These studies were performed in a variety of cell lines, both normal and transformed, that exhibited a range of receptor expression levels; they were performed with two different ligands (EGF and TGFα); and they were performed in the presence and absence of serum to control for environmental factors. We went to great lengths to ensure that these observations are not isolated phenomena, but are indeed intrinsic to the receptor, independent of its immediate environment.

It is unclear whether the average concentration of EGF in any tissue ever approaches the concentration required to activate low-affinity receptors. However, in cases of autocrine signaling, the effective concentration of EGFR ligands in the immediate vicinity of cell-surface receptors likely exceeds this threshold. Squamous cell carcinomas of the head and neck often rely on the activation of Stat3 for proliferation and survival [24]. Stat3 activity in these cancer cells has been shown to depend on autocrine activation of EGFR by secreted TGFα [24], [25]. The fact that Stat3 is only activated by low-affinity receptors in every cell type that we examined suggests that *in vivo* concentrations of EGFR ligands can stimulate low-affinity receptors and identifies a possible role for low-affinity receptors in the *in vivo* signaling of cancer cells.

Very recently, structural studies of the extracellular ligand-binding domain of *Drosophila* EGFR have supported negative cooperativity in ligand binding [26]. The authors showed that the first ligand binds with high affinity and induces a conformational change that promotes asymmetry in the dimer. The conformational change restrains the vacant binding site such that its affinity for binding the second ligand is reduced. High- and low-affinity binding sites therefore occur in the same receptor dimer and result from negative cooperativity rather than from distinct populations of receptor. The authors further argue that the second binding event must compromise either ligand-receptor or receptor-receptor contacts, and that therefore a doubly occupied dimer could have different interactions and signaling properties than a singly occupied one. Although this asymmetry has not been observed in the extracellular domain of human EGFR, a similar mode of regulation remains possible. If high- and low-affinity interactions do arise from negative cooperativity, singly-occupied dimers should be most abundant at low concentrations of ligand, and doubly occupied dimers should only form at higher concentrations of ligand. It is therefore possible that the Stat proteins and PLCγ1 can only be activated by doubly occupied dimers that have altered specificity, autophosphorylation or interactions.

EGFR has been extensively studied over the past three decades, and several recent analyses have provided system-level views and models of signaling downstream of the receptor [27]–[30]. These studies, however, have not accounted for the distinct signaling properties of high- and low-affinity receptors. In addition to the biological implications, our findings should benefit computational efforts to model this signaling network and predict cellular outcomes in response to diverse stimuli.
